# Supplementary material for: Dementia with Lewy bodies post-mortem brains reveal differentially methylated CpG sites with biomarker potential
Source: Commun Biol. 2022 Nov 22;5:1279. doi: 10.1038/s42003-022-03965-x (PMC9684551; doi:10.1038/s42003-022-03965-x)
Supplement: Supplementary file 2 — Supplementary Information [file 42003_2022_3965_MOESM2_ESM.pdf]

Supplementary Information

Dementia with Lewy bodies post-mortem brains reveal differentially methylated CpG sites with biomarker potential

Xiaojian Shao[1*], Sangeetha Vishweswaraiah[2], Miroslava Čuperlović-Culf[1,3,4], Ali Yilmaz[2,5], Celia Greenwood[6,7,8], Anuradha Surendra[1], Bernadette McGuinness[9], Peter Passmore[9], Patrick G. Kehoe[10], Michael E. Maddens[2,5], Steffany A.L. Bennett[3,4], Brian D. Green[11], Uppala Radhakrishna[2,5], Stewart F. Graham[2,5]*

[1] National Research Council of Canada, Digital Technologies Research Centre, Ottawa, Canada
[2] Oakland University-William Beaumont School of Medicine, Rochester, MI 48309, USA
[3] Ottawa Institute of Systems Biology, Ottawa, Ontario, Canada
[4] Department of Biochemistry, Microbiology, sand Immunology, Faculty of Medicine, University of Ottawa, Ottawa, Ontario, Canada
[5] Beaumont Research Institute, Royal Oak, MI 48073, USA
[6] Lady Davis Institute for Medical Research, Jewish General Hospital, Montréal, Canada
[7] Department of Epidemiology, Biostatistics and Occupational Health, McGill University, Montréal, Canada
[8] Department of Human Genetics, McGill University, Montréal, Canada
[9] Centre for Public Health, School of Medicine, Dentistry and Biomedical Sciences, Queen's University Belfast, Belfast, UK
[10] Dementia Research Group, Translational Health Sciences, Bristol Medical School, University of Bristol, Bristol, UK
[11] Institute for Global Food Security, School of Biological Sciences, Faculty of Medicine, Health and Life Sciences, Queen's University Belfast, Northern Ireland, UK

***Correspondence:**
Xiaojian Shao
Email: xiaojian.shao@nrc-cnrc.gc.ca

Stewart Graham
Email: stewart.graham@beaumont.edu

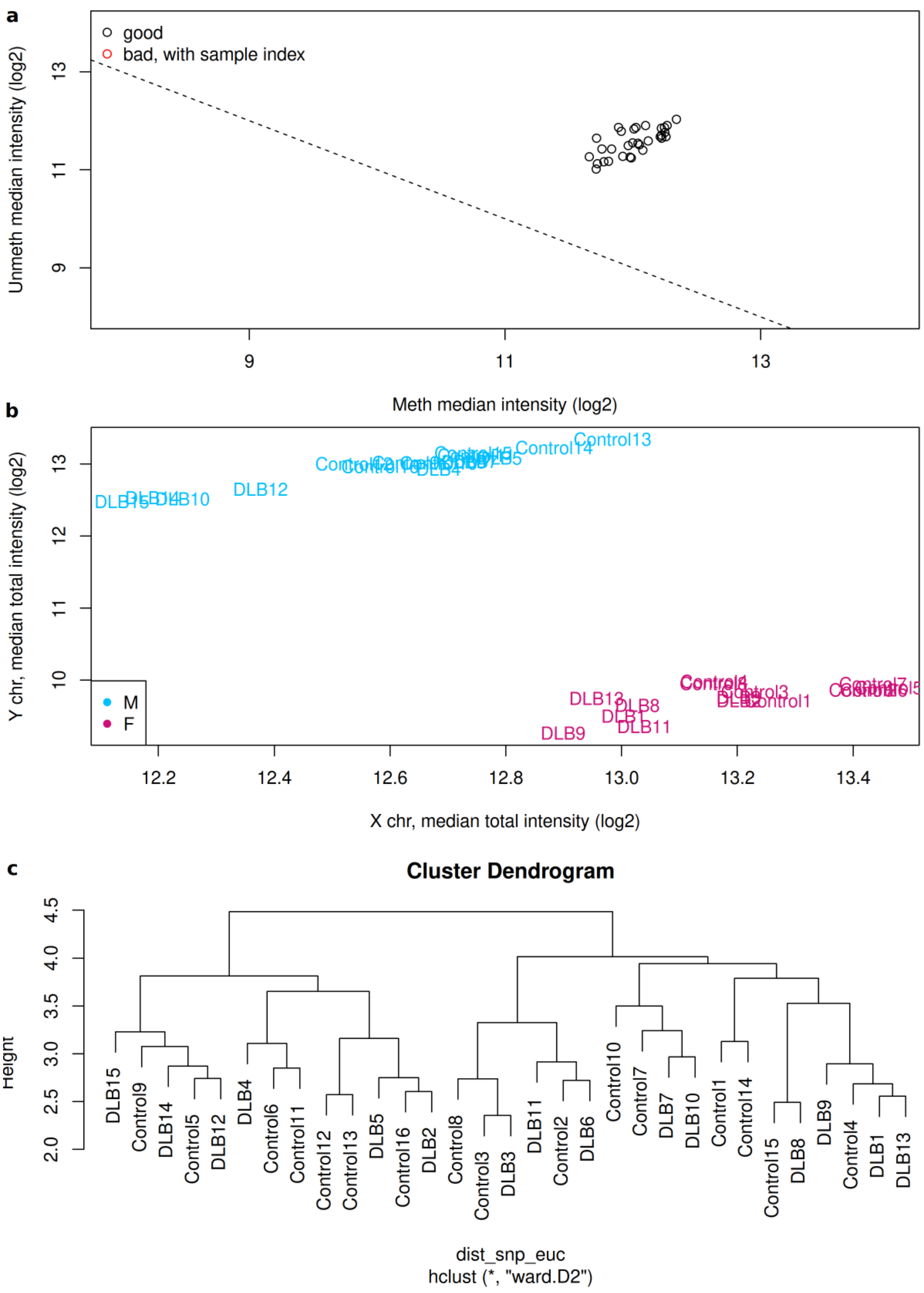

Supplementary Figure 1. Quality control of DLB EPIC array data. a Signal intensity plot of all the samples. Median intensity of methylated and unmethylated channels intensity per sample was calculated. **b** Sex prediction based on the median total intensity on sex-chromosomes. **c** Hierarchical clustering of samples with SNP profiles.

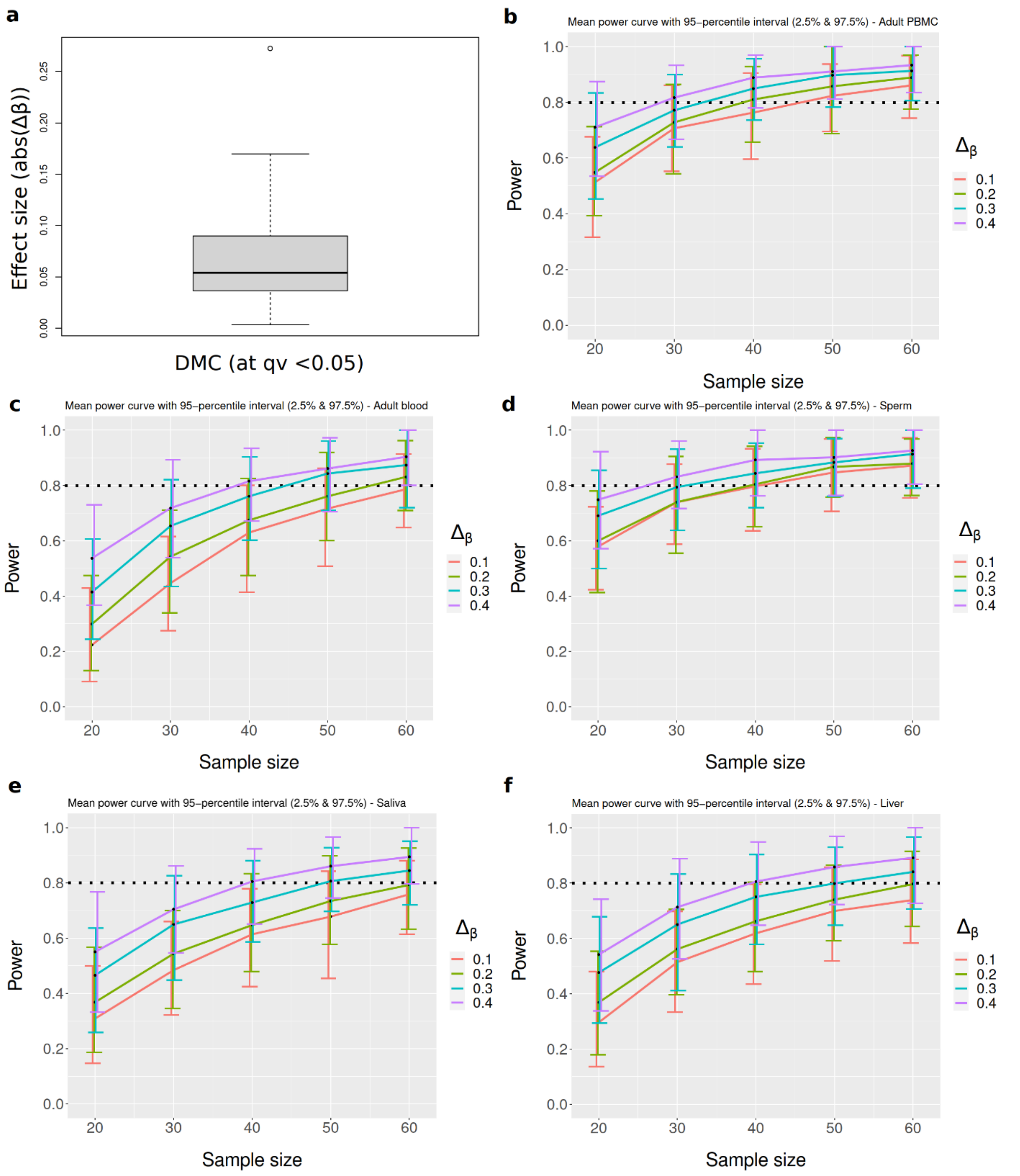

Supplementary Figure 2. Effect size and power calculation. a Effect size (Δβ) distribution of DLB-associated DMCs at q-value < 0.05. Power estimation against sample size with different effect sizes using pwrEWAS with different tissues as reference tissue methylome: **b** Adult PBMC; **c** Adult blood; **d** Sperm; **e** Saliva and **f** Liver. The 95-percentile intervals were included.

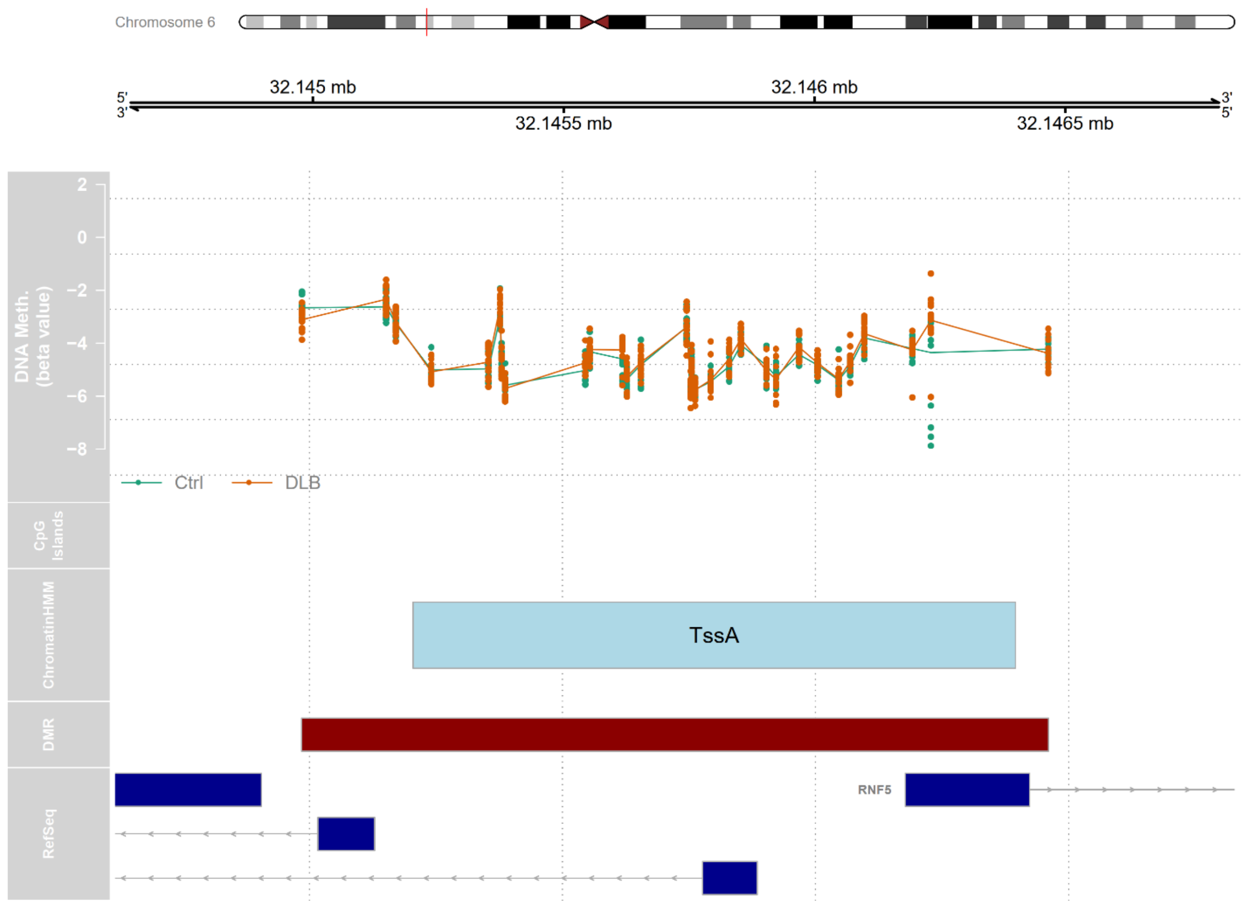

Supplementary Figure 3. **Example of DLB-associated DMR.** Example of one of the top DMR (a hyper-methylated region associated with the *RNF5* and *AGPAT1*) at q-value < 0.1.

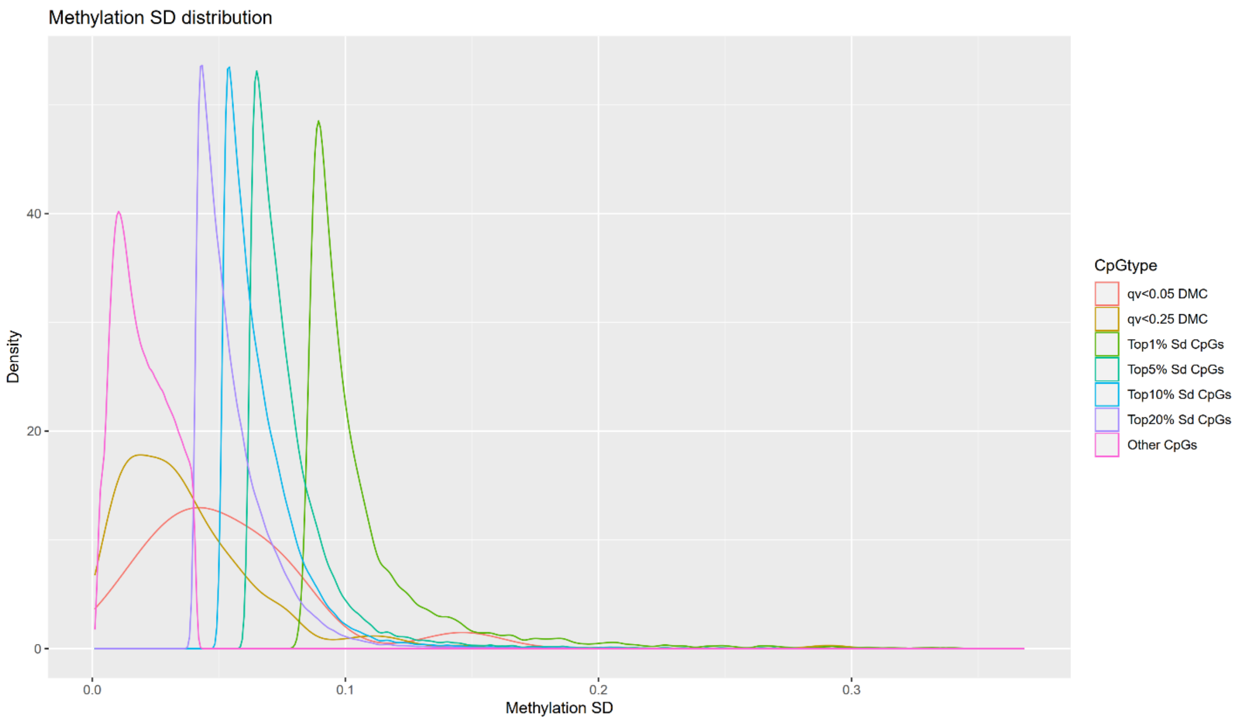

Supplementary Figure 4. The standard deviation (SD) distribution of DLB associated DMCs and top variable CpGs. DMCs with q-value < 0.05 and q-value < 0.25 were included. Top variable CpGs were grouped based on their SD intervals.

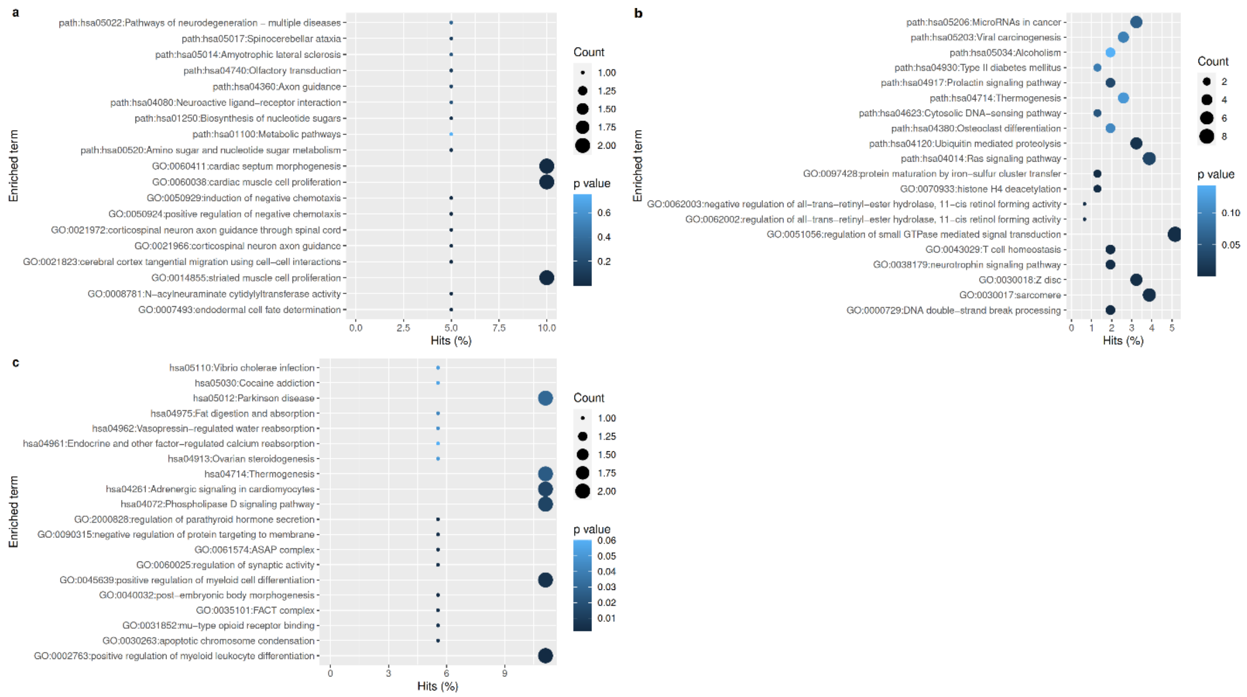

Supplementary Figure 5. **Annotation of DLB-associated DMCs and DMRs**. a Functional enrichment analysis of the q-value < 0.05 DMC-related genes. **b** Functional enrichment analysis of the q-value < 0.25 DMC-related genes. **c** Functional enrichment analysis of the q-value < 0.25 DMRs-related genes. The functional enrichment analysis on the DMCs and DMRs associated genes were performed using GOmeth and GOregion functions in the R package missMethyl. P-values were indicated by the colors while the counts of hits were indicated by the circle sizes.

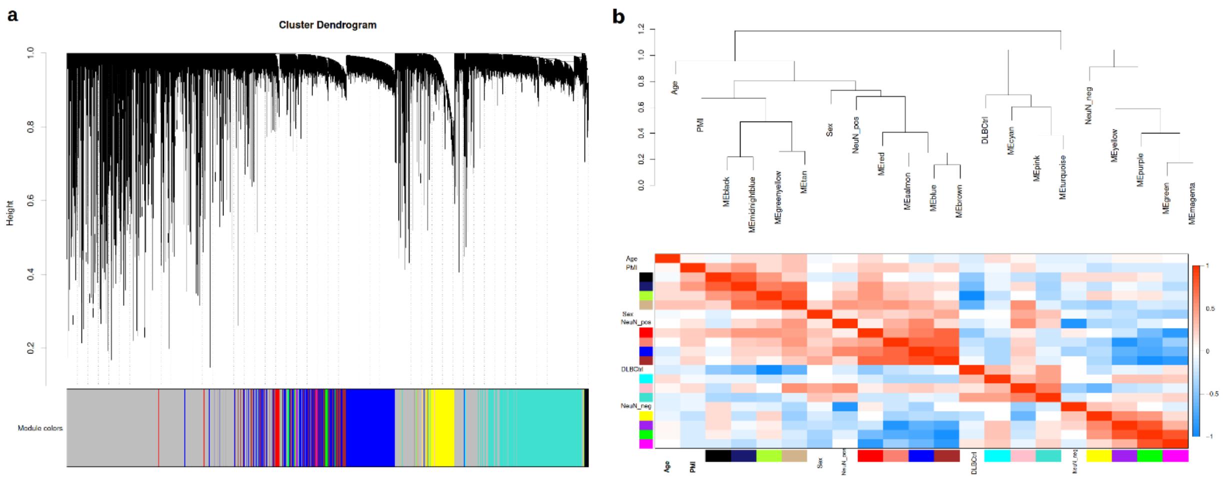

Supplementary Figure 6. **Weighted gene co-expression network analysis (WGCNA). A** Clustering and modules identified by WGCNA. **b** Trait correlation against modules identified in the WGCNA analysis.

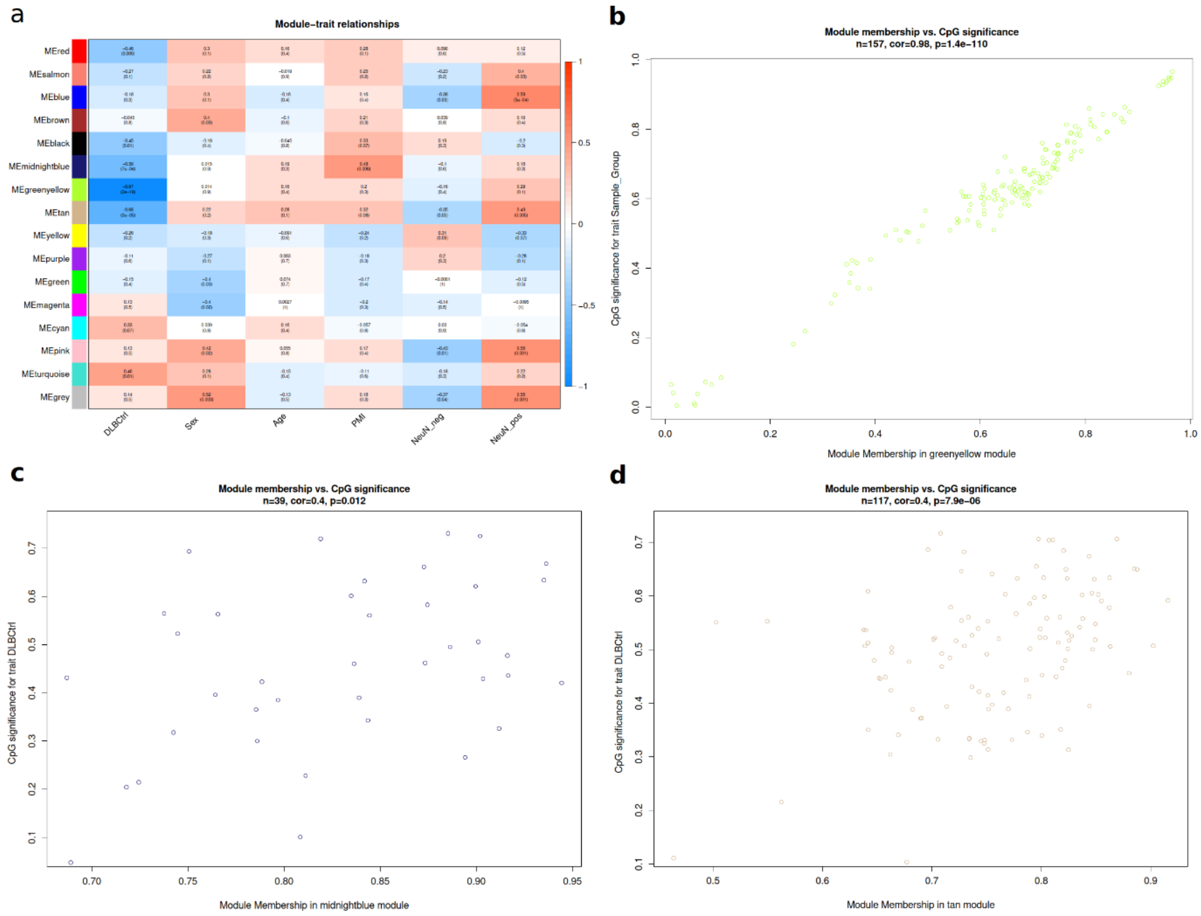

Supplementary Figure 7. **CpG Modules detected using WGCNA. a** Module-trait relationship heatmap as analysed using WGCNA. **b** Scatterplot of module membership and CpG significance for greenyellow module. **c** Scatterplot of module membership and CpG significance for mignightblue module. **d** Scatterplot of module membership and CpG significance for tan module.

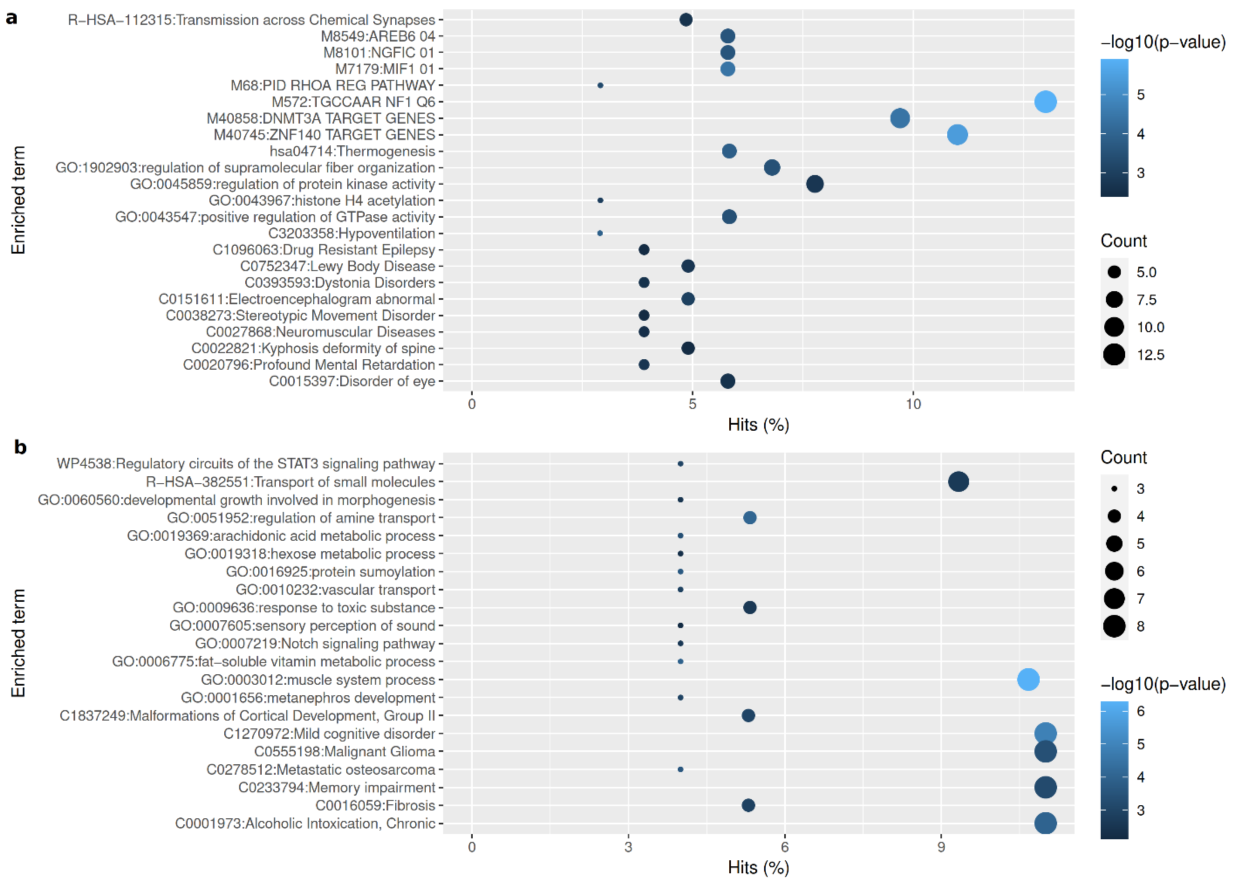

Supplementary Figure 8. **Annotation of the CpG modules identified with WGCNA. a** Functional enrichment and DisGeNET analysis of the greenyellow module-related genes using Metascape. **b** Functional enrichment and DisGeNET analysis of the tan module-related genes using Metascape. P-values were indicated by the colors while the counts of hits were indicated by the circle sizes.

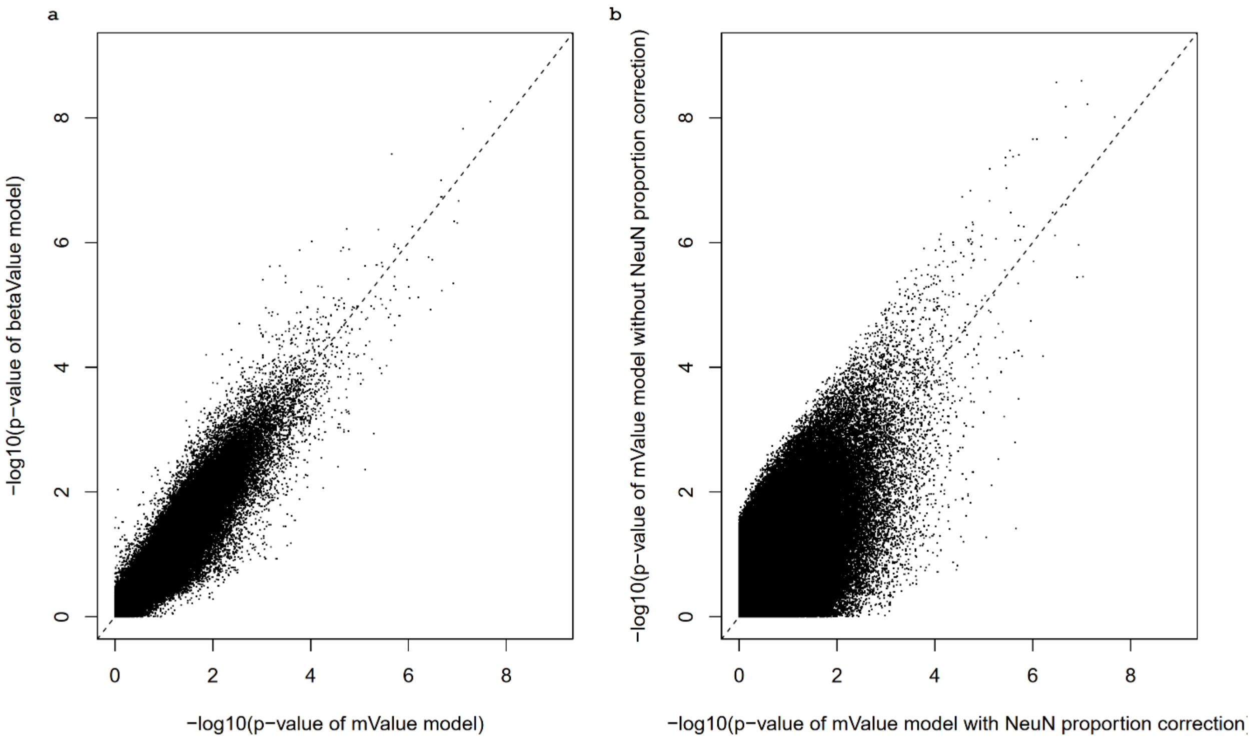

Supplementary Figure 9. **Scatterplots of the comparisons between different models. a** Scatterplot of the -log10(p-values) between the m-Value model and the beta-Value model. **b** Scatterplot of the -log10(p-values) between the m-Value models with and without NeuN proportion correction.

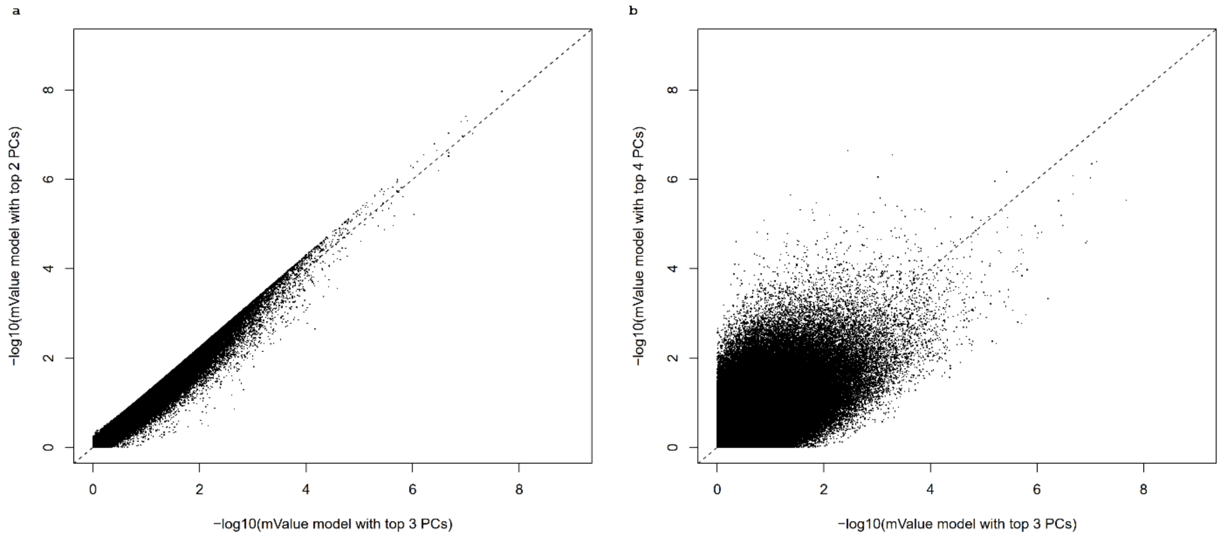

Supplementary Figure 10. **Scatterplots of the comparisons between m-Value models with different number of PCs. a** Scatterplot of the -log10(p-values) between the m-Value models corrected by top2 PCs and top3 PCs. **b** Scatterplot of the -log10(p-values) between the m-Value models corrected by top3 PCs and top4 PCs.

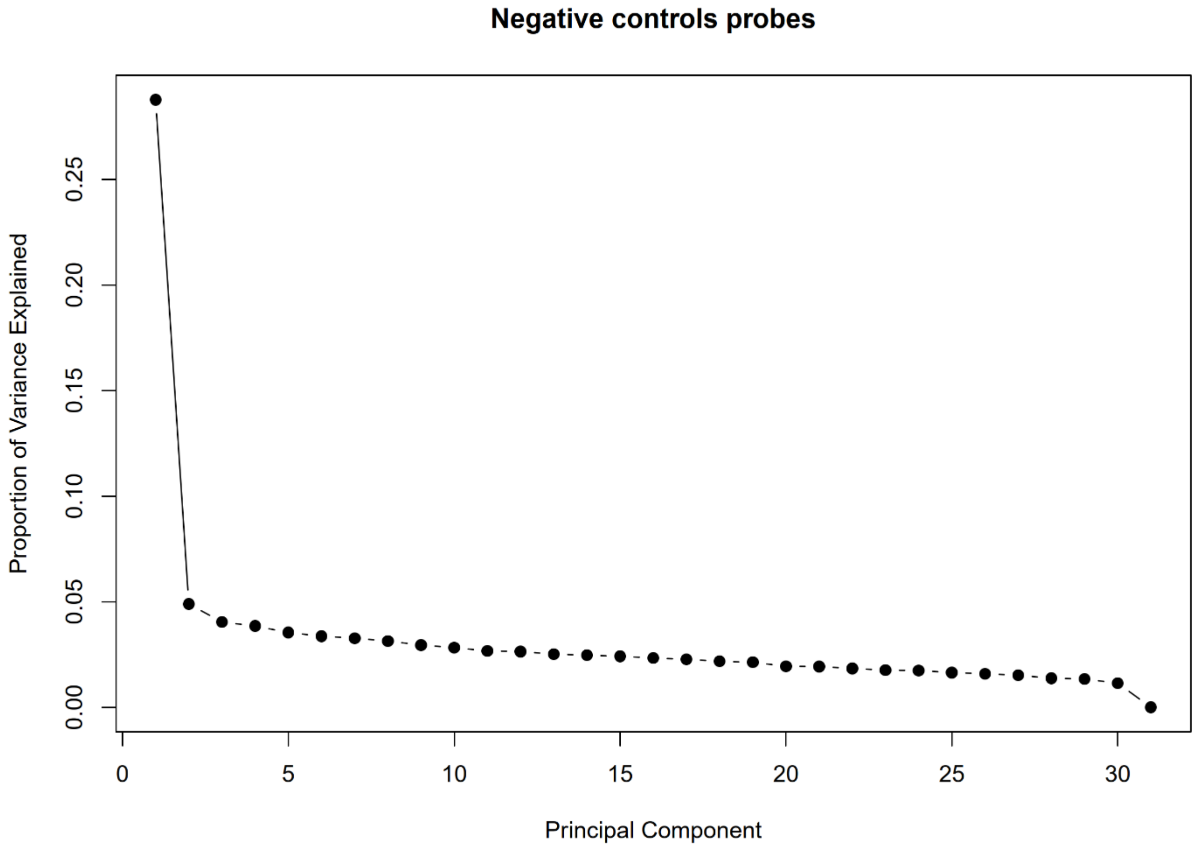

Supplementary Figure 11. Scree plot of the negative control probe-based PCA analysis.

Supplementary Table 1. Demographics of the samples involved in this study.

Sample name	Sex	Age (year)	PMI (hour)	Braak Stage
Control1	F	73	59	1
Control2	F	83	24	2
Control3	F	82	37	2
Control4	F	72	24	0
Control5	F	87	47	2
Control6	F	86	32	2
Control7	F	73	50	2
Control8	F	90	41	2
Control9	M	80	67	3
Control10	M	89	91	2
Control11	M	78	48	1
Control12	M	76	23	2
Control13	M	87	24	2
Control14	M	85	30	2
Control15	M	77	42	1
Control16	M	92	34.25	2
DLB1	F	81	81	3
DLB2	F	81	44	4
DLB3	F	97	24	2
DLB4	M	76	26	0
DLB5	M	86	15.25	3
DLB6	M	75	53	0
DLB7	M	78	75	2
DLB8	F	67	20	0
DLB9	F	76	33	3
DLB10	M	77	21	3
DLB11	F	79	26	2
DLB12	M	69	38	0
DLB13	F	90	42	2
DLB14	M	74	18	Diffuse cortical
DLB15	M	88	34.5	Diffuse cortical
