# Supplementary material for: Dementia with Lewy bodies post-mortem brains reveal differentially methylated CpG sites with biomarker potential
Source: Commun Biol. 2022 Nov 22;5:1279. doi: 10.1038/s42003-022-03965-x (PMC9684551; doi:10.1038/s42003-022-03965-x)
Supplement: Supplementary file 3 — Description of Additional Supplementary Files [file 42003_2022_3965_MOESM3_ESM.pdf]

Description of Additional Supplementary Files

File name: Supplementary Data 1
Description: The list of differentially methylated CpGs at FDR q-value < 0.1.

File name: Supplementary Data 2
Description: Lists of CpGs involved in different modules inferred with WGCNA. The annotated CpGs with greenyellow, midnightblue and tan modules are indicated in different spreadsheets.

File name: Supplementary Data 3
Description: The list of differentially methylated CpGs inferred with m-Value model at nominal p-value < 1e-3.

File name: Supplementary Data 4
Description: The list of differentially methylated CpGs inferred with beta-value model at nominal p-value < 1e-3.

File name: Supplementary Data 5
Description: The source data underlying the Figure 1.

File name: Supplementary Data 6
Description: The source data underlying the Figure 2.

File name: Supplementary Data 7
Description: The source data underlying the Figure 3-5.
