# Supplementary material for: Dementia with Lewy bodies post-mortem brains reveal differentially methylated CpG sites with biomarker potential
Source: Commun Biol. 2022 Nov 22;5:1279. doi: 10.1038/s42003-022-03965-x (PMC9684551; doi:10.1038/s42003-022-03965-x)
Supplement: Supplementary file 11 — Reporting Summary [file 42003_2022_3965_MOESM11_ESM.pdf]

nature portfolio

Corresponding author(s): Xiaojian Shao & Stewart Graham

Last updated by author(s): Aug 8, 2022

Reporting Summary

Nature Portfolio wishes to improve the reproducibility of the work that we publish. This form provides structure for consistency and transparency in reporting. For further information on Nature Portfolio policies, see our Editorial Policies and the Editorial Policy Checklist.

Statistics

For all statistical analyses, confirm that the following items are present in the figure legend, table legend, main text, or Methods section.

n/a	Confirmed	
☐	☒	The exact sample size (*n*) for each experimental group/condition, given as a discrete number and unit of measurement
☐	☒	A statement on whether measurements were taken from distinct samples or whether the same sample was measured repeatedly
☐	☒	The statistical test(s) used AND whether they are one- or two-sided<br>*Only common tests should be described solely by name; describe more complex techniques in the Methods section.*
☐	☒	A description of all covariates tested
☐	☒	A description of any assumptions or corrections, such as tests of normality and adjustment for multiple comparisons
☐	☒	A full description of the statistical parameters including central tendency (e.g. means) or other basic estimates (e.g. regression coefficient) AND variation (e.g. standard deviation) or associated estimates of uncertainty (e.g. confidence intervals)
☐	☒	For null hypothesis testing, the test statistic (e.g. *F*, *t*, *r*) with confidence intervals, effect sizes, degrees of freedom and *P* value noted<br>*Give P values as exact values whenever suitable.*
☒	☐	For Bayesian analysis, information on the choice of priors and Markov chain Monte Carlo settings
☒	☐	For hierarchical and complex designs, identification of the appropriate level for tests and full reporting of outcomes
☐	☒	Estimates of effect sizes (e.g. Cohen's *d*, Pearson's *r*), indicating how they were calculated

Our web collection on statistics for biologists contains articles on many of the points above.

Software and code

Policy information about availability of computer code

Data collection	We generated the DNA methylation array data using Illumina Infinium MethylationEPIC BeadChip arrays. We followed the Illumina protocol to perform the assay and followed by BeadChip imaging using Illumina iScan (Illumina, California, USA).
Data analysis	The Illumina EPIC array raw intensity data was processed and analyzed using the methylation analysis R package Minfi (version 1.36.0, R/Bioconductor). The neuronal proportions were estimated using R package estimateCellCounts with the setting for compositeCellType=DLPFC and with the reference data FlowSorted.DLPFC.450k. The differential methylation analysis between DLBs and controls were performed using linear regression modeling, implemented in limma (version 3.46.0). The differentially methylated regions were analyzed using DMRcate (version 2.8.0). The weighted gene co-expression network analysis was performed using the WGCNA package (version 1.69) in R. The analysis code could be accessed through https://github.com/xshaonrc/DLBepigenetics.

For manuscripts utilizing custom algorithms or software that are central to the research but not yet described in published literature, software must be made available to editors and reviewers. We strongly encourage code deposition in a community repository (e.g. GitHub). See the Nature Portfolio guidelines for submitting code & software for further information.

Data

Policy information about availability of data

All manuscripts must include a data availability statement. This statement should provide the following information, where applicable:

- Accession codes, unique identifiers, or web links for publicly available datasets
- A description of any restrictions on data availability
- For clinical datasets or third party data, please ensure that the statement adheres to our policy

The datasets generated during and/or analyzed during the current study are available in the GEO repository (GSE190348).

Human research participants

Policy information about studies involving human research participants and Sex and Gender in Research.

Reporting on sex and gender	This study used the term sex for analysis. The study collected both male and female patients and used sex as a co-variate in our data analysis. This study collected 31 patients in total. There are 8 males and 7 females in DLB group, and 8 males and 8 females in control group. The sex of each sample was determined by a statistical model and was predicted by using the getSex() function in the Minfi R library (version 1.36.0, R/Bioconductor). The results should applied to both male and female.
Population characteristics	This study collected 15 pathologist confirmed DLB cases and 16 non-cognitively impaired controls from a cohort of the Brains for Dementia Research (BDR) Initiative. These samples were sex and age matched. There are 8 males and 7 females in DLB group, and 8 males and 8 females in control group. The average age of DLB patients was 79.6 years and 81.9 years for DLB and control groups respectively. Genotype analysis revealed there is not outliers in our study.
Recruitment	These patients were part of BDR initiative. Participants were recruited with support from ARUK and AS (both press teams and lay representatives), using national and local press, TV and radio coverage, articles in charity newsletters, national magazines with an older following, BDR posters, leaflets, memory clinics, talks at carer/support groups, Women's Institute, University of the Third Age. BDR has a dedicated website with links from the funding charities, MRC and Human Tissue Authority websites. All potential donor recruitment and clinic assessments are undertaken in compliance with the Mental Capacity Act (2005) and other good clinical practice guidelines. More details please refer to PMID: 30452415 (Francis, P. T. et al. Journal of Alzheimer's Disease 2018).
Ethics oversight	This study was approved by the Beaumont Health System's Human Investigation Committee (HIC No.: 2018-387).

Note that full information on the approval of the study protocol must also be provided in the manuscript.

Field-specific reporting

Please select the one below that is the best fit for your research. If you are not sure, read the appropriate sections before making your selection.

☒ Life sciences ☐ Behavioural & social sciences ☐ Ecological, evolutionary & environmental sciences

For a reference copy of the document with all sections, see nature.com/documents/nr-reporting-summary-flat.pdf

Life sciences study design

All studies must disclose on these points even when the disclosure is negative.

Sample size	We collected post-mortem brain tissue samples (harvested from the neocortex, Brodmann area 7) from pathologist confirmed DLB cases (n=15) and non-cognitively impaired controls (n=16) from a cohort of the Brains for Dementia Research (BDR) Initiative. The effect size underline the DLB disease is largely unknown. However, we have tried a R package "pwrEWAS", which was designed to estimate the power specific to EWAS studies, to perform the power analysis. Based on this estimation, our sample in this study is slightly underpowered.
Data exclusions	There were no data excluded from the original analysis.
Replication	While quite limited studies have been conducted on the analysis of DLB vs control epigenetic profiles with large-scale samples, a few DLB-associated genes were reported through different DNA methylation studies and were summarized in a recent review paper. We observed signals at 6 of 10 noted genes (60%) in our study. We also compared the results from a more recent study by Pihlstrøm et al. but none of their signals were replicated in our study. This may due to the different brain tissue regions, and the different disease stages explored in the two studies. We don't have a replication cohort analyzed for this study.
Randomization	The samples were randomly obtained from a cohort of the Brains for Dementia Research (BDR) Initiative.
Blinding	The investigators were blinded to group allocation during data collection and analysis.

Reporting for specific materials, systems and methods

We require information from authors about some types of materials, experimental systems and methods used in many studies. Here, indicate whether each material, system or method listed is relevant to your study. If you are not sure if a list item applies to your research, read the appropriate section before selecting a response.

Materials & experimental systems

n/a	Involved in the study
☒	☐ Antibodies
☒	☐ Eukaryotic cell lines
☒	☐ Palaeontology and archaeology
☒	☐ Animals and other organisms
☒	☐ Clinical data
☒	☐ Dual use research of concern

Methods

n/a	Involved in the study
☒	☐ ChIP-seq
☒	☐ Flow cytometry
☒	☐ MRI-based neuroimaging
